# A Fast Evaluation Method for Electrical Performance of Frequency and Pattern Reconfigurable Microstrip Antenna Based on Electromechanical Coupling

**DOI:** 10.3390/mi13091412

**Published:** 2022-08-27

**Authors:** Pengying Xu, Xiaoxian Xu, Kabin Lin, Rong Yu, Daxing Zhang, Yan Wang, Zhihai Wang, Kunpeng Yu, Wenzhi Wu, Xiaofei Ma, Congsi Wang

**Affiliations:** 1 Key Laboratory of Electronic Equipment Structure Design, Ministry of Education, Xidian University, Xi’an 710071, China; 2 Guangzhou Institute of Technology, Xidian University, Guangzhou 510555, China; 3 School of Information and Control Engineering, Xi’an University of Architecture and Technology, Xi’an 710055, China; 4 Zhejiang Fengfan CNC Machinery Co., Ltd., Huzhou 313113, China; 5 CETC No. 38 Research Institute, Hefei 230088, China; 6 Xi’an Institute of Space Radio Technology, Xi’an 710100, China

**Keywords:** reconfigurable microstrip antenna, resonance frequency coupling model, pattern function coupling model

## Abstract

With the constant increase in communication requirements in modern society, the number and type of antennas on communication platforms have been increasing at an accelerating rate. This has led to a continuous increase in platform volume and weight, and the electromagnetic environment of antenna operating has increasingly worsened, seriously restricting the further development of communication systems. As a new communication system antenna type, a reconfigurable microstrip antenna can reconstruct operating frequencies, beam directions, etc., by changing the antenna structure to provide the good multifunction characteristics of a single antenna, avoiding the electromagnetic compatibility issues caused by numerous system antennas. At present, most of the research on reconfigurable antennas judges the influence of structural characteristics on electromagnetic characteristics by simulation, which has imposed restrictions on their development and application. Therefore, a reconfigurable antenna with a resonant frequency of 8.66 GHz and 15.26 GHz and a reconfigurable antenna with maximum radiation directions of 36.2° and −36.5° are designed in this paper, and the electromechanical coupling theory of the reconfigurable antennas is studied. The resonance frequency coupling model and the pattern function coupling model considering the structural deformation of a reconfigurable microstrip antenna are established. Within the applicable range of antenna structural parameters, the relative error between the resonance frequency coupling model and the pattern function coupling model is less than 5%, which meets practical engineering application requirements. Finally, the method is shown by experimentation to verify the accuracy and validity of the proposed electromechanical coupling model.

## 1. Introduction

With the development of 5G and 6G wireless communication systems, the number and type of antennas on communication platforms are increasing, and frequency spectrum resources are increasingly being used, which makes platform structures more complex and massive. The electromagnetic compatibility between antennas becomes increasingly prominent, which seriously restricts the further upgrading of communication systems. The concept of a “reconfigurable antenna” has emerged and developed rapidly. Reconfigurable microstrip antennas are widely used in wireless communication due to their broadband, multifunction, lightweight, and low electromagnetic interference capabilities. As a new type of antenna, a reconfigurable microstrip antenna can not only dynamically change the antenna structure, change the antenna operating frequency, and significantly increase the antenna operating bandwidth but also enable the antenna to quickly and dynamically adapt to the performance requirements under different conditions so that the designed microstrip antenna can provide the multifunctionality of a single antenna; thus, the number, volume, and weight of antennas are greatly reduced and effectively expand antenna bandwidth, and the electromagnetic compatibility problem caused by a large number of antennas in a system is avoided [1,2,3,4].

Compared with traditional antenna structures, the electrical performance of a reconfigurable antenna is more dependent on its complex mechanical structure. A reconfigurable antenna structure contains switching devices and corresponding control circuits, and the structural characteristics of many structural combinations directly determine the antenna’s electromagnetic performance [5,6]. The structural characteristics of a reconfigurable antenna can include many parameters, such as the shape, size, position, and working state of each component in the antenna structure. Any change in the structural parameters can affect the performance of the reconfigurable antenna from the desired design value. Therefore, a high-performance broadband multifunctional reconfigurable antenna can only be designed from the point of view of electromechanical coupling [7,8] and interdisciplinarity. However, at present, the design of reconfigurable antennas depends on engineering experience, and their performance characteristics are changed by adjusting current distributions on antenna elements or arrays by means of mechanical adjustability [9,10], PIN diodes [11,12,13], photoelectric switches [14,15], and MEMS switches [16,17,18]. The influence of structural characteristics on electromagnetic characteristics is only judged by simulation because of the lack of systematic research theory. Therefore, it is necessary to study the electromechanical coupling theory of reconfigurable antennas.

Similar to traditional antennas, the theory of electromechanical coupling of reconfigurable microstrip antennas mainly refers to the coupling relationship between the reconfigurable antenna structure field and electromagnetic field, which requires establishing a mathematical model (also called the electromechanical coupling model) between reconfigurable antenna structural parameters and electrical performance, studying the influence rule of structural parameters on electrical performance, and analyzing the disturbance mechanism of reconfigurable antenna electrical performance in a complex environment. At present, the research on electromechanical coupling theory is quite mature, and many scholars at home and abroad have conducted numerous studies. HSC Wang [19] assumed two possible deformations of a phased array antenna: bending and bowl deformations. Based on this, the influence of antenna structural deformation on electric performance was analyzed. Considering random errors during antenna installation, the electromechanical coupling theory of phased array antennas was studied by combining the errors. Zulich P [20] analyzed the influence of antenna array deformation on antenna gain and ground moving target indication for low orbit L-band spaceborne microstrip array antennas. Geng J P studied the relationship between the bending angle and the electrical performance of a microstrip antenna with a special structure when the antenna is bent. It was found that the antenna can still operate normally when the bending angle of the antenna is within a certain range. Bae-Ian Wu [21] studied the change in electrical performance of a microstrip array antenna under the condition of array deformation and found that the irregular deformation of the array antenna has a great influence on the electrical performance of the array antenna, but when the phase of the exciting current of each element patch is adjusted to a certain extent, the electrical performance of the antenna tends to improve. From the point of view of mechatronics coupling, scholars have extensively researched the influences of array antenna structural deformations, random errors, service environments and other factors on electrical performance and achieved certain theoretical results [22,23,24,25,26,27,28,29]. Mao J [25] analyzed the coupling mechanism between the serving environment and electrical performance of microstrip array antennas and studied the change in electrical performance when the antenna element undergoes different degrees of bending deformation, based on which they proposed a coupling model of bending deformation–gain loss for array antennas, realizing the electromechanical co-optimization of satellite-borne microstrip array antennas in thermal environments. Han [26] analyzed the electromechanical coupling model of a microstrip antenna considering bending deformation and the influence of thermal deformation of the structure on the electrical performance of a satellite-borne microstrip antenna array; an electromechanical coupling model of a microstrip antenna element based on its structural deformation was established, and the change in electrical performance of a microstrip antenna element under a severe service environment was analyzed based on the electromechanical coupling model.

The above research on electromechanical coupling theory was mainly focused on phased array antennas and traditional microstrip array antennas. Reconfigurable antennas, as a new type of antenna, are mostly focused on structural design. Software simulation or numerical analysis methods are used to analyze their electrical performance. However, few people have studied the coupling relationship and influence mechanism between the structural parameters and electrical performance of reconfigurable antennas. To better study the design method of pattern reconfigurable antennas, Guo [30] proposed a method for calculating the radiation pattern of reconfigurable antennas based on the vector superposition of the space electric field, clarified the coupling model for calculating the electric performance of antennas, and simply verified it with MATLAB software. However, the coupling relationship between reconfigurable antenna structure and electrical performance has not been extensively studied, and the influence of structural parameters on antenna electrical performance has not been analyzed. Yang [31] proposed a compact-coupled array antenna based on reconfigurable technology, combined a phased array antenna with a reconfigurable principle, designed an antenna unit and feed structure, and carried out physical tests and verification. However, this paper only designed a reconfigurable antenna unit based on the principle of array coupling and formed an array. The coupling relationship between the reconfigurable antenna structure and electromagnetic characteristics was not qualitatively analyzed.

In this paper, a frequency reconfigurable microstrip antenna and a pattern reconfigurable pattern microstrip antenna are designed. Based on the reconfigurable principle and the analysis theory of rectangular microstrip antennas, a fast prediction method for the resonant frequency of reconfigurable antennas based on transmission line model theory and equivalent current paths and a radiation pattern calculation method based on cavity model theory and mutual coupling effects are proposed, and a 3 × 3 reconfigurable antenna electromechanical coupling model is established. Including the antenna structure-resonant frequency coupling model and structure-radiation pattern coupling model, the frequency characteristics and radiation performance of reconfigurable antennas under different switching combinations can be quickly predicted, and the influence of antenna structure parameters on the performance in a certain range can be analyzed. The results show that within the applicable range, the relative errors of calculation and simulation are less than 5%, which can quickly assess the changes in antenna electrical performance under different environmental loads and further guide the design of reconfigurable antenna structures.

## 2. Establishment of an Electromechanical Coupling Model for a Frequency and Pattern Reconfigurable Microstrip Antenna

**Figure 1:**
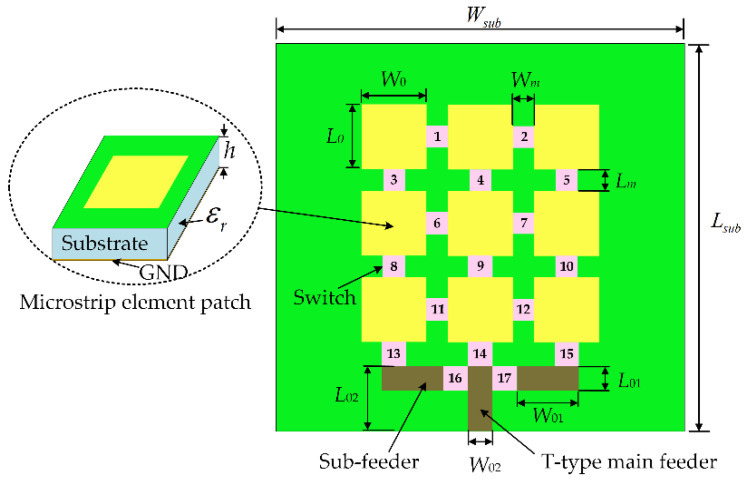
Schematic of a reconfigurable microstrip antenna.

**Figure 2:**
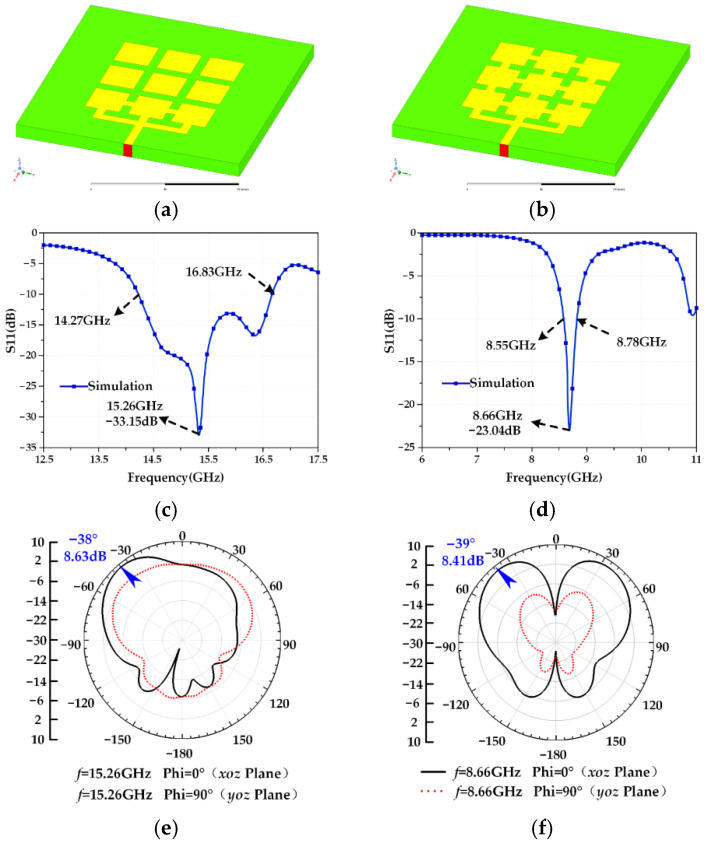
(**a**) Mode 1 antenna structure; (**b**) Mode 2 antenna structure; (**c**) S11 curve of Mode 1 antenna; (**d**) S11 curve of Mode 2 antenna; (**e**) radiation pattern of Mode 1 antenna; (**f**) radiation pattern of Mode 2 antenna.

**Figure 3:**
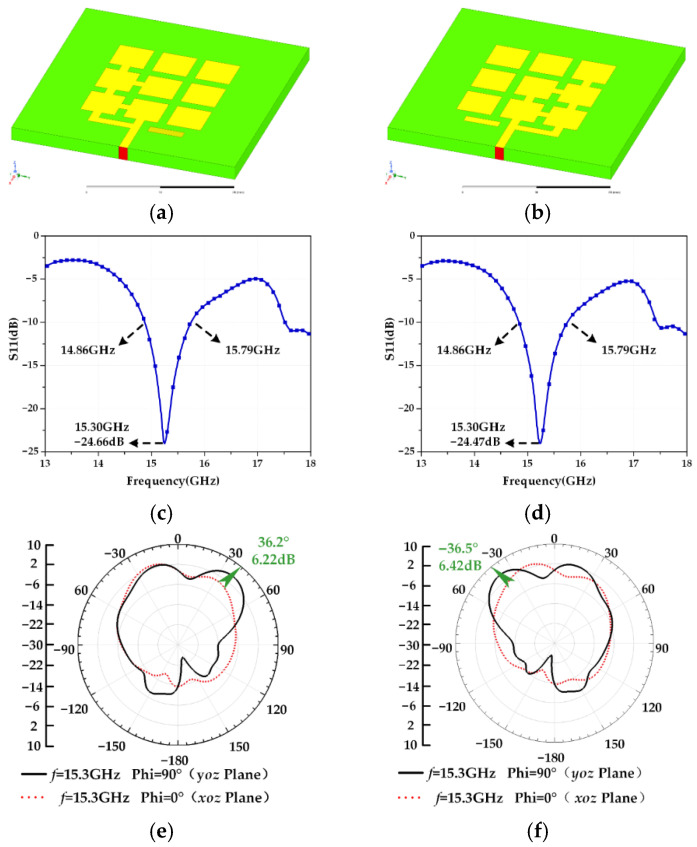
(**a**) Mode 3 antenna structure; (**b**) Mode 4 antenna structure; (**c**) S11 curve of Mode 3 antenna; (**d**) S11 curve of Mode 4 antenna; (**e**) radiation pattern of Mode 3 antenna; (**f**) radiation pattern of Mode 4 antenna.

A reconfigurable microstrip antenna structure is proposed in this paper, as shown in Figure 1. The reconfigurable microstrip antenna has 17 switches, 2^17^ switch states. The structural parameters of each part of the antenna are shown in Table 1. Based on this structure, a frequency reconfigurable antenna and a pattern reconfigurable antenna are designed using an adaptive immune annealing algorithm [32], and the structure and electrical performance are shown in Figure 2 and Figure 3. When the antenna is in mode 1 and mode 2, frequency reconfiguration can be realized. When the antenna is in mode 3 and mode 4, pattern reconfiguration can be realized. Table 2 summarizes the electrical performance of the antenna in mode 1–4. The patch element uses copper material, and the dielectric substrate is made of Rogers RT/Duroid 5880. The relative permittivity is 2.2, the dielectric loss is low, and its electrical performance changes very little with frequency. The bottom surface of the microstrip array antenna is covered with a copper layer. There is a longstanding problem in designing a reconfigurable microstrip antenna based on the optimization algorithm: electromagnetic simulation software will spend considerable time in the electromagnetic simulation calculation of the antenna. Even adopting a more efficient optimization algorithm cannot effectively reduce the time for optimizing antenna design. Therefore, this paper uses the antennas shown in Figure 2 and Figure 3 to establish an electromechanical coupling model for antenna electrical performance calculation, which can better meet the real-time evaluation requirements of antenna performance.

As seen in Figure 2, the maximum radiation directions of the Mode 1 antenna and the Mode 2 antenna on the *xoz* plane are −38° and −39°, respectively, and the beam coverages are similar. Therefore, the Mode 1 antenna and the Mode 2 antenna exhibit similar pattern performance to meet the design requirements of the frequency reconfigurable antenna. The Mode 1 antenna and Mode 2 antenna can be reconfigured between 8.55–8.78 GHz and 14.27–16.83 GHz with an approximately unchanged radiation direction (−38° and −39°); the S11 parameters are less than −10 dB, and gain is greater than 5 dB in the operating frequency band, which indicates that the antenna has good radiation capability and meets the requirements for a general communication system.

According to Figure 3, the radiation patterns of the Mode 3 antenna and Mode 4 antenna in the *yoz* plane are symmetrical, with maximum radiation directions of 36.2° and −36.5°, respectively, and the beam coverage is basically symmetrical. The operating frequency bands of both antennas are 14.86–15.79 GHz, which meets the design requirements of a pattern reconfigurable antenna. The Mode 3 antenna and the Mode 4 antenna can be reconstructed in the *yoz* plane with an operating band of 14.86–15.79 GHz (Ku-band) unchanged for +36.2° and −36.5°. The gain of both antennas is greater than 5 dB, and S11 is less than −10 dB in the working frequency band, which indicates that the antennas have good matching characteristics and can be applied in the field of satellite communication.

### 2.1. Ideal Reconfigurable Microstrip Antenna Electromechanical Coupling Model

#### 2.1.1. Antenna Resonance Frequency Coupling Model Based on Transmission Line Model Theory and Equivalent Current Distribution

**Figure 4:**
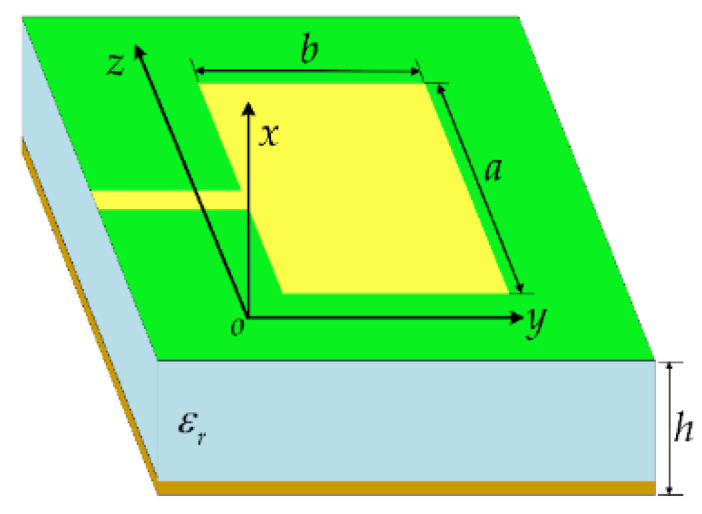
Rectangular microstrip antenna.

The general structure of a rectangular microstrip antenna is shown in Figure 4. Based on the transmission line model, D.L. Sengupta [33] derived an improved formula for calculating the resonant frequency of a rectangular microstrip antenna. As shown in Equation (1), the length of the microstrip antenna is *b,* and the width is *a*.

(1) $$f=\frac{\beta c}{2\pi \sqrt{\varepsilon_{e}}}=\frac{c}{2L\sqrt{\varepsilon_{e}}}\left[\frac{1-\xi }{1+\xi \ln\left(1.123L\sqrt{\varepsilon_{e}}\right)/h}\right]$$

where,

(2) $$\xi =\frac{2h}{\pi \tau \varepsilon_{e}b}$$

(3) $$\varepsilon_{e}=\frac{1}{2}\left[\varepsilon_{r}+1+\left(\varepsilon_{r}-1\right){\left(1+\frac{10h}{a}\right)}^{-1/2}\right]$$

(4) $$\xi =\frac{2h}{\pi \tau \varepsilon_{e}b}\tau =\frac{h}{a}\left[\frac{a}{h}+1.393+0.667\ln\left(\frac{a}{h}+1.444\right)\right]$$

**Figure 5:**
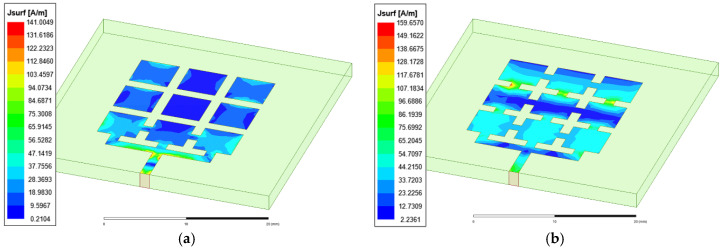
(**a**) Mode 1 antenna surface current distribution; (**b**) Mode 2 antenna surface current distribution.

Antenna radiation generates an electromagnetic field with specific performance, and the basic condition for producing electromagnetic radiation is a time-varying current. For the solution of the radiation field of any antenna, only the current distribution on the surface of the patch must be known, and the radiation characteristics of the antenna can be obtained by integrating the current element. Therefore, determining the antenna radiated electromagnetic field essentially requires the distributed current on the antenna, but even if the current or voltage of the feed port and the antenna structure are known, it is not easy to determine the current distribution on the antenna, which is an internal antenna problem. To address the difficult distribution of antenna current, combined with the calculation model of microstrip antenna resonant frequency derived from the transmission line theory of the microstrip antenna, we believe that if the equivalent length and width of surface current distribution on the microstrip antenna array can be obtained, it is not difficult to calculate the resonant frequency of the antenna, and electromagnetic numerical analysis software can visually display the current distribution on each position of the antenna structure. Therefore, based on an analysis of the surface current distribution of the antenna by electromagnetic simulation software, the equivalent length and width of the surface current distribution of the array microstrip antenna can be obtained. Taking a frequency reconfigurable antenna (Mode 1 antenna and Mode 2 antenna) as an example, electromagnetic simulation analysis is carried out, and the current distribution is shown in Figure 5.

When the antenna is in working Mode 1, by analyzing the surface current of the antenna, it is considered that the equivalent circuit path of the antenna is *L*_0_ in length b and 3*W*_0_ in width a; these values can be placed into Equation (1). The solution equation of the resonant frequency and the resolving result of resonant frequency are shown in Table 3. The HFSS simulation results are very close to the theoretical calculation results, and the method is considered effective.

Based on an analysis of the surface current of the Mode 2 antenna, it is found that the current on patches 4, 5, and 6 is mainly distributed at the edge of the patch, and there is almost no current distribution in the middle of the patch. Therefore, the equivalent path length of the reconfigurable antenna should be less than its physical length. It is considered that the equivalent circuit path length b of the antenna is 2*L_0_* and the width a is 3*W_0_*; these values are then placed into Equation (1), the resonant frequency is shown in Table 4. The HFSS simulation results are in accordance with the theoretical calculation results.

From the above analysis, it can be concluded that for reconfigurable microstrip antennas with different switching states, only one full-wave simulation is required to obtain their resonant frequency characteristics and surface current distribution. Then, we can calculate the length and width of the surface patch and introduce it into our reconfigurable microstrip antenna structural parameter–resonant frequency coupling model. Then, the frequency characteristics of the antenna can be quickly calculated within a certain range, which is of great value for engineering applications.

#### 2.1.2. Antenna Pattern Coupling Model Based on Cavity Model Theory and the Mutual Coupling Effect

With the enhancement of antenna function, the structural design of antennas has become increasingly complex, especially in the field of reconfigurable microstrip antennas, and the number of radiation patches is usually more than one. The reconfigurable antenna studied in this paper consists of nine identical rectangular small patch elements that are placed together on the surface of a dielectric substrate to form a coplanar structure along with a cavity with a metal ground plate under the dielectric substrate. In this way, several cavities are placed in close proximity in parallel in the same medium space. A coupling effect also occurs between adjacent metal patches. In general, the antenna elements are far away from each other, and the coupling effect between them can be neglected. However, a reconfigurable antenna connects each patch via a switch. The distance between patches is very close, and the mutual coupling effect is obvious, so it must be considered. Based on cavity model theory [34,35], by observing and analyzing the current and electric field distributions of reconfigurable antennas and considering the coupling effect between patches, a method for calculating the reconfigurable antenna pattern function is proposed, and its structural parameters and direction map function are established and verified.

#### 2.1.3. Field Distribution Calculation of the Patch Element Based on the Cavity Model

**Figure 6:**
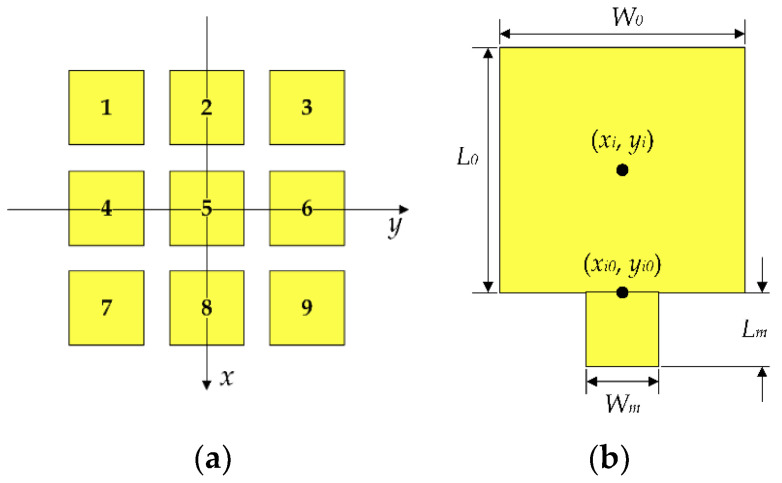
(**a**) Reconfigurable microstrip antenna coordinate system and patch number; (**b**) coordinate system of the *i*th patch element.

Figure 6 shows a structural sketch of the frequency and pattern reconfigurable microstrip antenna proposed in this paper. When determining the pattern, the influence of the feeder is ignored temporarily, and the reconfigurable antenna can be regarded as 9 rectangular microstrip patch elements. The pattern function of the reconfigurable antenna is analyzed based on cavity model theory.

The space between each patch element and the ground plate is considered a resonant cavity, which is surrounded by a magnetic wall, and the upper and lower sides are electric walls. According to the operating principle of the rectangular microstrip antenna, the equivalent magnetic current exists on the four walls of the cavity, and the radiation field of the antenna can be calculated from the equivalent magnetic current around the cavity. A coordinate system is set up as shown in Figure 6. Each patch element is numbered as shown in Figure 6a. If the center coordinate of the *i*th patch element is set as (*x_i_*, *y_i_*), then the electric field distribution of any patch element numbered *i* is:

(5) $$\left\{\begin{array}{l} E_{i\theta }=\frac{4k_{0}hB_{imn}}{\lambda_{0}R}e^{-jk_{0}R}e^{j\left(x_{i}\frac{u}{L_{0}}+y_{i}\frac{v}{W_{0}}+\frac{m\pi +n\pi }{2}\right)}\sin\frac{u+m\pi }{2}\sin\frac{v+n\pi }{2}\\ \;\;⦁\;\left[\frac{L_{0}{}^{2}}{u^{2}-{\left(m\pi \right)}^{2}}+\frac{W_{0}{}^{2}}{v^{2}-{\left(n\pi \right)}^{2}}\right]\sin\theta \sin\varphi \cos\varphi \\ E_{\iota \varphi }=\frac{4k_{0}hB_{imn}}{\lambda_{0}R}e^{-jk_{0}R}e^{j\left(x_{i}\frac{u}{L_{0}}+y_{i}\frac{v}{W_{0}}+\frac{m\pi +n\pi }{2}\right)}\sin\frac{u+m\pi }{2}\sin\frac{v+n\pi }{2}\\ \;\;⦁\;\left[\frac{L_{0}{}^{2}\cos^{2}\varphi }{u^{2}-{\left(m\pi \right)}^{2}}+\frac{W_{0}{}^{2}\sin^{2}\varphi }{v^{2}-{\left(n\pi \right)}^{2}}\right]\sin\theta \cos\theta \end{array}\right.$$

where,

(6) $$E_{iz}=\sum_{m,n}B_{imn}\cos\frac{m\pi }{L_{0}}\left(x+\frac{L_{0}}{2}-x_{i}\right)\cos\frac{n\pi }{W_{0}}\left(y+\frac{W_{0}}{2}-y_{i}\right)$$

(7) $$B_{imn}=jk_{0}\eta_{0}I_{i0}\frac{\frac{\delta_{om}\delta_{on}}{W_{0}L_{0}}}{k^{2}-k_{mn}^{2}}\cos\frac{m\pi x_{i0}}{L_{0}}\cos\frac{n\pi y_{i0}}{W_{0}}j_{0}\left(\frac{m\pi W_{m}}{2W_{0}}\right)$$

(8) $$\left\{\begin{array}{c} u=k_{0}L_{0}\sin\theta \cos\varphi \\ v=k_{0}W_{0}\sin\theta \sin\varphi \end{array}\right.$$

*I_i_*_0_ is the feed current of each patch, and when the MEMS switch is closed, it can be equivalent to a feeder-to-feed patch element of a reconfigurable antenna. *B_imn_* is a different excitation mode. For the microstrip antenna studied in this paper, there are two different excitation modes TM01 and TM10, corresponding to *B_i_*_01_ and *B_i_*_10,_ respectively.

From the above analysis, it is known that only the excitation current *I_i_*_0_ for each patch is required and *B_imn_* for each patch excitation mode is determined, i.e., the values of *m* and *n* in Equations (6) and (7), and then the electric field distribution of each small patch unit is obtained by Equation (5).

#### 2.1.4. Reconfigurable Antenna Pattern Function Coupling Model Considering Mutual Coupling

Through theoretical analysis, based on the field strength function of each patch element, the field strength function of a reconfigurable antenna under a certain combination of switches can be obtained, and then the pattern function of a reconfigurable antenna can be obtained. For the Mode 3 antenna, patches 7 and 8 are direct feeding elements, patches 1, 4, and 5 are indirect feeding elements, and patches 3, 6, and 9 are parasitic elements. By observing the current distribution on the surface of the antenna element, it is found that there is also current on the parasitic patch because the small distance between the patches leads to enhanced coupling between the patches, and an induced current is generated on the parasitic patch.

#### 2.1.5. Solution of Patch Excitation Current

**Figure 7:**
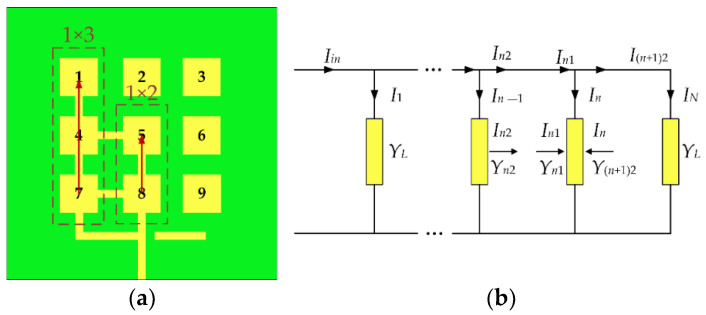
(**a**) Schematic of the Mode 3 antenna series-fed patch antenna array; (**b**) equivalent circuit diagram of the series-fed patch antenna array.

We know that patches 1, 4, 5, 7, and 8 are feeder patches in the reconfigurable microstrip antenna, while the feeders feed at the 7 and 8 patches, temporarily ignoring the parasitic patches. The remaining patches can be equivalent to two series-fed patch antenna arrays (1 × 2, 1 × 3) to calculate the feeding current, as shown in Figure 7a. Figure 7b shows the equivalent circuit diagram of a series-fed antenna array.

According to ref. [36], the admittance and excitation currents of each element in the series-fed antenna array are:

(9) $$Y_{n2}=\frac{\left(Y_{L}+Y_{(n+1)2})(e^{\alpha dx}+e^{-\alpha dx})+Y_{0}(e^{\alpha dx}-e^{-\alpha dx}\right)}{Y_{0}(e^{\alpha dx}+e^{-\alpha dx})+(Y_{L}+Y_{(n+1)2})(e^{\alpha dx}-e^{-\alpha dx})}$$

(10) $$I_{n-1}=\frac{I_{n}}{2}[(e^{\alpha dx}+e^{-\alpha dx})+\frac{Y_{L}+Y_{(n+1)2}}{Y_{0}}(e^{\alpha dx}-e^{-\alpha dx})]$$

where $\alpha =\frac{0.11513\times A}{d_{x}}$, *Y_L_* is the input admittance for the patch element, *Y*_0_ is the characteristic admittance of the equivalent transmission line, *A* is the loss of the microstrip line, and *dx* = *L*_0_ + *L*_m_ is the distance between the patch elements.

Assuming the initial feeding current *I*_7_ = *I*_8_= 1, the current of each feeding patch element (*I*_4_, *I*_5_, *I*_1_) can be obtained from Equations (9) and (10) in turn. At the same time, it is considered that there is no exciting current on the parasitic patch; only the induced current is caused by the coupling effect between the patches. Let the mutual coupling coefficient be:

(11) $$S_{ij}=\left\{\begin{array}{l} 1+S_{ij}\;i=j\\ S_{ij}\;\;i\neq j \end{array}\right.$$

The mutual coupling coefficient between patches is extracted from HFSS, and the current on each patch is:

(12) $$I_{i0}{}^{\prime }=I_{i0}+\overset{N}{\underset{j=0}{\sum }}S_{ij}\cdot I_{j0}$$

The calculated current for each patch is shown in Table 5:

- Patch element excitation mode judgment

**Figure 8:**
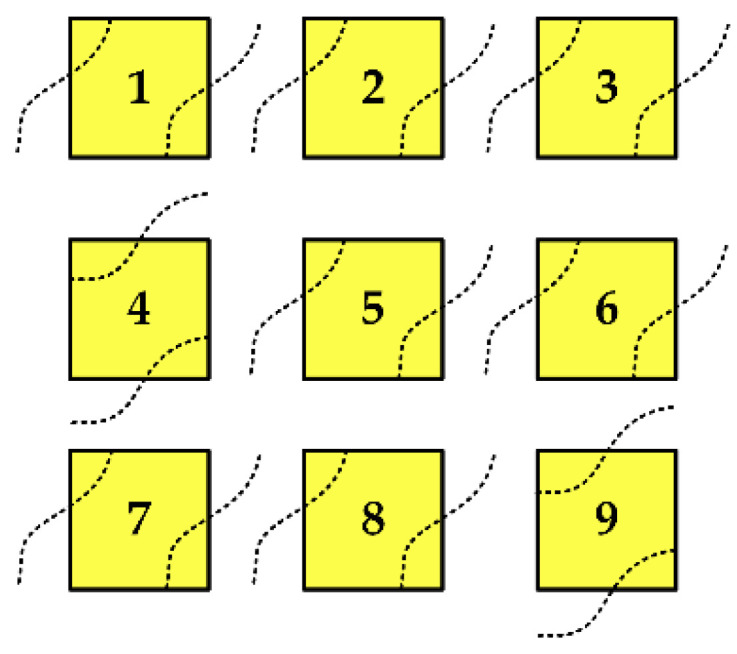
Electric field distribution of the reconfigurable microstrip antenna.

Due to the close distance between the patch elements connected by the MEMS switch, the excitation mode of the patch cannot be unified to the TM01 or TM10 mode as the traditional rectangular patch element. The excitation modes of each patch element of the reconfigurable microstrip antenna could potentially be different, but it is difficult to directly determine or calculate the operation mode of the patch element in theory at present. By observing the electric field distribution at the edge of the patch element after HFSS simulation, we obtain a more intuitive field distribution diagram according to cavity model theory and the actual electric field distribution diagram, as shown in Figure 8. It can be seen that patches 1, 2, 3, 5, 6, 7, and 8 work in the TM10 model and patches 4 and 9 work in the TM01 model.

*B_imn_* is obtained by substituting the excitation current and mode of each patch into Equation (7), and the field strength *E_iz_* of each patch element is obtained by substituting it into Equation (6). By sorting and removing the R-related constant terms in Equation (5) and superimposing all the field strengths of the patch elements, the pattern function of the reconstructed antenna in plane E and H can be obtained from the following equation:

(13) $$F_{E}\left(\theta \right)=\underset{i}{\sum }V_{i10}e^{j\left(y_{i}k_{0}\;sin\;\theta \right)}F_{1}\left(\theta \right)+\underset{j}{\sum }V_{j01}e^{j\left(y_{i}k_{0}\;sin\;\theta \right)}F_{2}\left(\theta \right),\;i\;=1,2,3,5,6,7,8;\;\;j=4,9$$

(14) $$F_{E}\left(\theta \right)=\underset{i}{\sum }V_{i10}e^{j\left(y_{i}k_{0}\;sin\theta \;\right)}F_{1}\left(\theta \right)+\underset{j}{\sum }V_{j01}e^{j\left(y_{i}k_{0}\;sin\theta \;\right)}F_{2}\left(\theta \right),\;i\;=1,2,3,5,6,7,8;\;\;j=4,9$$

where,

(15) $$F_{1}\left(\theta \right)=\frac{sin(\frac{k_{0}W_{0}\;sin\;\theta }{2})}{\frac{k_{0}W_{0}\;sin\;\theta }{2}}cos\;\theta$$

(16) $$F_{2}(\theta )=\cos(\frac{k_{0}L_{0}\;\sin\;\theta }{2})$$

**Figure 9:**
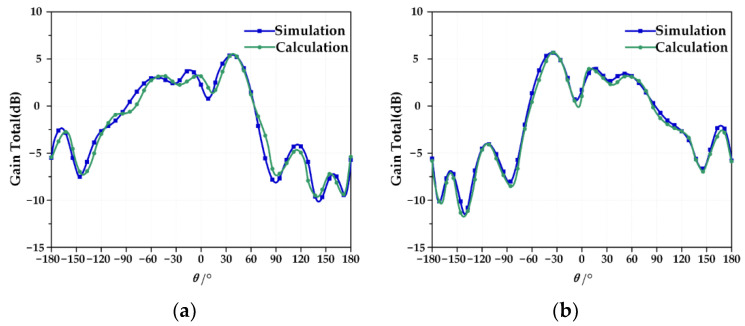
(**a**) Comparison of equation calculation and simulation results of Mode 3 antenna; (**b**) Comparison of equation calculation and simulation results of Mode 4 antenna.

The calculation results of the pattern coupling model are in good agreement with the HFSS simulation, and the reconfigurable antenna pattern is shown in Figure 9a. The beam directions are +36° and +36.2°, which shows that the method proposed in this paper is effective.

When the antenna is in Mode 4, its current distribution is completely symmetrical with Mode 3, as is the field distribution. The pattern of the antenna is:

(17) $$F_{E}\left(\theta \right)=\underset{i}{\sum }V_{i10}e^{j\left(y_{i}k_{0}\;sin\;\theta \right)}F_{1}\left(\theta \right)+\underset{j}{\sum }V_{j01}e^{j\left(y_{i}k_{0}\;sin\;\theta \right)}F_{2}\left(\theta \right),\;i\;=1,2,3,4,5,8,9;\;\;j=6,7$$

(18) $$F_{H}\left(\theta \right)=\underset{i}{\sum }V_{i10}e^{j\left(x_{i}k_{0}\;sin\;\theta \right)}F_{2}\left(\theta \right)+\underset{j}{\sum }V_{j01}e^{j\left(x_{i}k_{0}\;sin\;\theta \right)}F_{1}\left(\theta \right),\;i\;=1,2,3,4,5,8,9;\;\;j=6,7$$

The results of the pattern coupling equation of the reconfigurable antenna pattern agree well with the HFSS simulation; beam pointing is −36.4° and −36.5°, respectively, as shown in Figure 9b, which indicates the accuracy of the method in this paper.

### 2.2. Reconfigurable Microstrip Antenna Coupling Model Considering Structural Deformation

During the service of a reconfigurable antenna, external loads will lead to deformation of the antenna array and displacement of the element position, which will lead to problems such as gain reduction, side lobe elevation, and poor pointing accuracy, which can seriously restrict the performance of the reconfigurable antenna. Therefore, to rapidly predict the electrical performance of a reconfigurable antenna after deformation, the coupling model of a reconfigurable microstrip antenna considering structural deformation is analyzed and established based on the coupling model established in Section 2.1.

**Figure 10:**
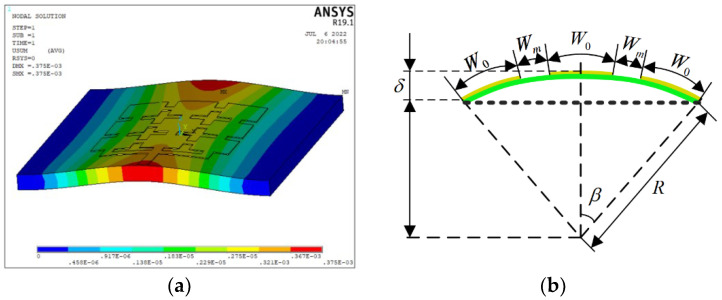
(**a**) Reconfigurable microstrip antenna deformation at an ambient temperature of 120 °C; (**b**) schematic diagram of the bending deformation of the reconfigurable antenna element.

A typical antenna service environment of space is selected to analyze the structural deformation of the reconfigurable microstrip antenna element. The space-borne microstrip array antenna is mainly subjected to long-term solar irradiation and a space low-temperature heat sink, with a fluctuation range of −160 °C to 140 °C. When affected by a thermal environment, the microstrip antenna structure is prone to thermal distortion, which affects the antenna electrical performance. In this paper, the range of the thermal deformation of the antenna is −0.5–0.5 mm by finite element simulation. Figure 10a shows the deformation of the antenna at a 120 °C extreme temperature. Here, the cubic polynomial is selected to fit the thermal deformation surface of the reconfigurable antenna element structure, and the deformation of the antenna structure can be approximated to that of the curved surface. Taking 120 °C as an example, the maximum deformation is 0.375 mm.

When both ends of the reconfigurable microstrip antenna are constrained, antenna distortion mainly occurs in the *x* direction. Based on the antenna deformation, an equivalent analysis of the bending deformation of the antenna element is carried out. Figure 10b shows a schematic diagram of the bending deformation of the antenna element. where *δ* is the bending deformation, which can be simulated and fitted by ANSYS software.

The following geometric relationships can be obtained from Figure 10b:

(19) $$R\beta =\frac{3W_{0}}{2}+W_{m}$$

(20) $$\delta =R\left(1-cos\;\beta \right)$$

where *R* is the bending radius, *δ* is the bending deformation, and *β* is the circumferential angle corresponding to the bending deformation.

According to the above equation, the relationship between bending deformation and reconfigurable antenna size *W*_0_ and *W_m_* is:

(21) $$\delta =\frac{\frac{3}{2}W_{0}+W_{m}}{\beta }\left(1-cos\;\beta \right)$$

(22) $$W_{m}{}^{\prime }=\frac{W_{m}\;sin\;\beta }{\beta }$$

(23) $$W_{0}{}^{\prime }=\frac{W_{0}\;sin\;\beta }{\beta }$$

With *W*_0_ = 4*W_m_*, the equivalent structural dimension after bending deformation is:

it can be obtained that the distance between the patch elements after deformation is $d_{x}{}^{\prime }=W_{0}{}^{\prime }+W_{m}{}^{\prime }$, by substituting $d_{x}{}^{\prime }$ into Equations (9)–(10), the excitation current $I_{ij}{}^{\prime }$ of all elements after deformation can be obtained in turn. The equivalent deformation $W_{0}{}^{\prime }$ is substituted into Equations (1)–(4) to obtain the antenna structural parameters-resonant frequency coupling model of the deformed Mode 1 antenna and Mode 2 antenna, as shown in Equations (24)–(26); by substituting the deformations $W_{0}{}^{\prime }$ and $I_{ij}{}^{\prime }$ into the Equations (13)–(14) and Equations (17)–(18), a deformed coupling model of the structure parameters- pattern function of the Mode 3 antenna and the Mode 4 antenna can be obtained, as shown in the Equations (27)–(30).

(24) $$f=\frac{\beta c}{2\pi \sqrt{\varepsilon_{e}}}=\frac{c}{2L\sqrt{\varepsilon_{e}}}\left[\frac{1-\xi }{1+\xi \ln\left(1.123L\sqrt{\varepsilon_{e}}\right)/h}\right]$$

where,

(25) $$\varepsilon_{e}=\frac{1}{2}\left[\varepsilon_{r}+1+\left(\varepsilon_{r}-1\right){\left(1+\frac{10h\beta }{W_{0}\;sin\;\beta }\right)}^{-1/2}\right]$$

(26) $$\tau =\frac{\beta h}{W_{0}\;sin\;\beta }\left[\frac{a}{h}+1.393+0.667\ln\left(\frac{W_{0}\;sin\;\beta }{\beta h}+1.444\right)\right]$$

(27) $$F_{E}\left(\theta \right)=\underset{i}{\sum }V_{i10}e^{j\left(y_{i}k_{0}\;sin\;\theta \right)}F_{1}\left(\theta \right)+\underset{j}{\sum }V_{j01}{}^{\prime }e^{j\left(y_{i}k_{0}\;sin\;\theta \right)}F_{2}\left(\theta \right)$$

(28) $$F_{E}\left(\theta \right)=\underset{i}{\sum }V_{i10}e^{j\left(y_{i}k_{0}\;sin\;\theta \right)}F_{1}\left(\theta \right)+\underset{j}{\sum }V_{j01}{}^{\prime }e^{j\left(y_{i}k_{0}\;sin\;\theta \right)}F_{2}\left(\theta \right)$$

where,

(29) $$F_{1}\left(\theta \right)=\frac{\;sin\;(\frac{k_{0}W_{0}\;sin\;\beta \;sin\;\theta }{2\beta })}{\frac{k_{0}W_{0}\;sin\;\beta \;sin\;\theta }{2\beta }}\;cos\theta$$

(30) $$F_{2}\left(\theta \right)=\;cos(\frac{k_{0}L_{0}\;sin\;\theta }{2})$$

### 2.3. Accuracy and Applicability Analysis of Reconfigurable Microstrip Antenna Coupling Model

For a reconfigurable microstrip antenna, the radiation characteristics of the antenna will change when the structural parameters change or errors exist. By changing the values of *W*_0_ and *L*_0_ at patch deformation, we compare the calculated results of the coupling model with the HFSS software simulation results to verify the accuracy of the reconfigurable microstrip antenna coupling model considering the structural deformation established in this paper and determine the applicable scope of the coupling model.

**Figure 11:**
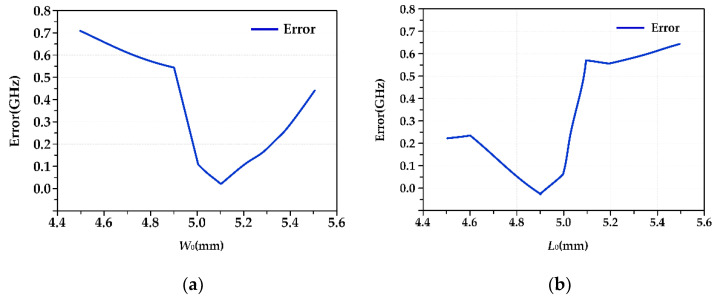
(**a**) Frequency calculation error (W0 parameter change); (**b**) frequency calculation error (L0 parameter change).

The antenna works at 15.26 GHz when it is in Mode 1. Table 6 shows the results of the HFSS simulation and coupling model calculation for different *W*_0_ parameters and *L*_0_ parameters. Figure 11a,b show the error (absolute value) distribution curves corresponding to the *W*_0_ parameters and *L*_0_ parameters.

In the range of 4.5 mm to 5.5 mm, based on W_0_ = 5 mm, the calculation error of the coupling model increases with increasing or decreasing *W*_0_. The maximum error is 0.71 GHz, the minimum error is 0.01 GHz, and the maximum relative error is 4.45%. Based on *L*_0_ = 5 mm, with the increase or decrease of *L*_0_, the error gradually increases, with the maximum error being 0.64 GHz, the minimum error being 0.02 GHz, and the maximum relative error being 4.32%. In addition, from the two error analysis figures, we can see the changes in the *W*_0_ parameters and *L*_0_ parameters. The errors of the coupling model calculation and simulation analysis results are symmetrically distributed. Based on these results, we can determine the law of error changes in future research to reduce errors and improve theoretical calculation accuracy.

**Figure 12:**
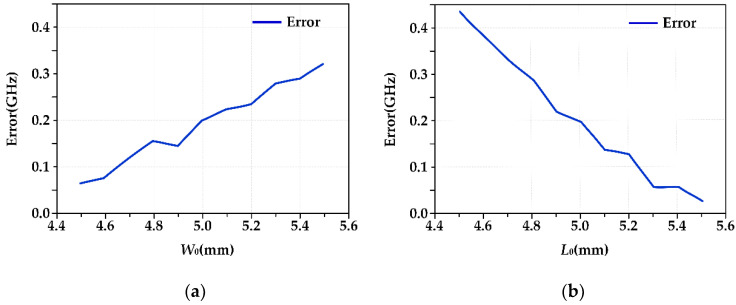
(**a**) Frequency calculation error (*W*_0_ parameter change); (**b**) frequency calculation error (*L*_0_ parameter change).

Similarly, when the antenna is in Mode 2, the working frequency is 8.66 GHz. The results of the HFSS simulation and coupling model calculation corresponding to different *W*_0_ and *L*_0_ parameters are shown in Table 7, and the corresponding error distribution curves are shown in Figure 12.

In the range of 4.5 mm to 5.5 mm, with the increase in *W*_0_, the calculation error of the coupling model gradually increases, with the maximum error being 0.31 GHz, the minimum error being 0.08 GHz, and the maximum relative error being 3.64%. With the increase in the *L*_0_ value, the error decreases gradually, with the maximum error being 0.43 GHz, the minimum error being 0.03 GHz and the maximum relative error being 4.65%. As with the Mode 1 antenna, the two error analysis diagrams show that when *W*_0_ and *L*_0_ change, the error calculated by the coupling model is symmetrically distributed.

When the antenna is in Mode 3, the reconfigurable beam direction is +36.2°. Table 8 shows the results of the HFSS simulation and coupling model calculation corresponding to different *W*_0_ and *L*_0_ parameters. Table 9 and Table 10 show the error results (absolute value) of the two methods.

When the *W*_0_ parameters change, the error between the results of the coupling model calculation and HFSS simulation results is significantly less than that when the *L*_0_ parameters change. When the *W*_0_ parameter changes, the maximum pointing error is 2.2°, the minimum pointing error is 0.2°, and the maximum relative error is 6.9%. When the *L*_0_ parameter changes, the error of the coupling model calculation is large in some cases. When the *L*_0_ parameter changes greatly, the beam pointing will change obviously, and the antenna will lose its reconfigurable characteristics. The reason may be that the coupling between the patches has changed due to the change in the *L*_0_ parameter, and the radiation mechanism of the antenna has also changed. The original analysis method is no longer applicable. However, it is considered that the coupling model is applicable and effective when the *W*_0_ parameter changes within 0.4 mm and *L*_0_ changes within 0.1 mm.

When the antenna is in Mode 4, the reconfigurable beam is pointing to −36.5°. The values of the *W*_0_ and *L*_0_ parameters are changed, the results of the HFSS simulation are compared with the coupling model calculation, and the calculation errors of the coupling model are analyzed, as shown in Table 11, Table 12 and Table 13.

When the *W*_0_ parameter changes, the error between the results of the coupling model calculation and HFSS simulation is significantly smaller than that of the *L*_0_ parameter. When the *W*_0_ parameter changes, the maximum pointing error is 3.1°, the minimum pointing error is 0.1°, and the maximum relative error is 9.8%. When the *L*_0_ parameter changes greatly, the beam pointing changes obviously, the antenna loses its reconfigurable characteristics, and the calculation error of the coupling model is large. The reason is that when the *L*_0_ parameter changes severely, the coupling between the patch elements changes, which results in a change in the radiation mechanism of the antenna. In general, the coupling model is applicable when the *W*_0_ parameter changes in the range of 0.4 mm and the *L*_0_ changes in the range of 0.1 mm.

## 3. Experimental Verification of the Electromechanical Coupling Model of a Reconfigurable Microstrip Antenna

To verify the electromechanical coupling model of the reconfigurable antenna established in the second part of this paper, experiments are used to verify the correctness and effectiveness of the electromechanical coupling model. The basic idea of the experiment is to use the external gravity load to deform the antenna structure, then measure the frequency characteristics and radiation performance of the antenna under different structural deformations, and then use the HFSS software and the electromechanical coupling model to calculate the antenna electrical performance under the same deformation. The electrical properties are compared with those measured experimentally to verify the correctness of the coupled model.

### 3.1. Experimental Scheme

**Figure 13:**
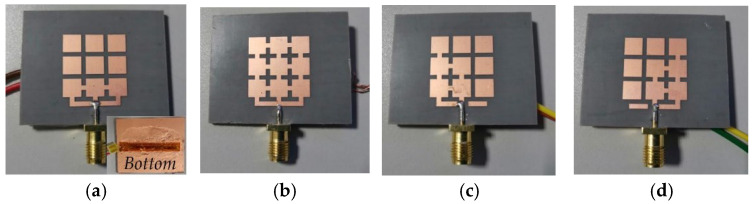
Physical prototypes of four reconfigurable antennas. (**a)** Mode 1 antenna; (**b**) Mode 2 antenna; (**c**) Mode 3 antenna; (**d**) Mode 4 antenna.

To verify the correctness and effectiveness of the electromechanical coupling model of the reconfigurable antenna, we designed a reconfigurable antenna prototype, as shown in Figure 13. The disconnection of the switch is realized by the presence or absence of copper sheets. The reconfigurable microstrip antenna unit structure includes radiation patches, dielectric substrates, ground plates, and SMA connectors. Among them, the radiation patches and ground plates are made of copper material; the dielectric substrate is made of Rogers RT/duroid 5880 material, which is soft and prone to deformation.

According to the analysis of the third part of this paper, the main environmental load of the spaceborne reconfigurable microstrip antenna is thermal load, and the thermal deformation of the antenna will lead to the deterioration of the electrical performance of the antenna. Due to the limited experimental conditions, it is impossible to effectively measure the change in the electrical properties of the reconfigurable microstrip antenna after deformation under thermal environmental loads. Therefore, the solution adopted is to design an equivalent deformation experiment scheme, that is, to use external force to deform the reconfigurable microstrip antenna, use high-precision resistance strain gauges to measure the deformation of the antenna structure, and use a vector network analyzer to measure the electrical performance parameters such as the resonant frequency and gain of the correspondingly deformed antenna.

**Figure 14:**
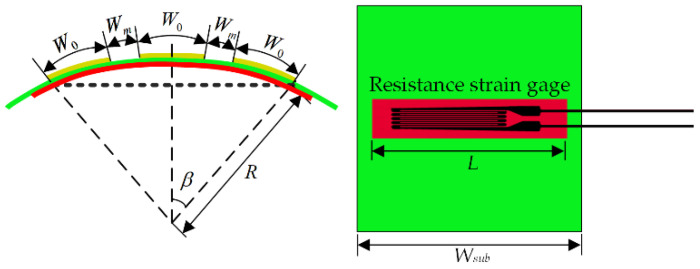
Bending deformation of the reconfigurable antenna element.

Figure 14 shows a schematic diagram of the bending deformation of the reconfigurable antenna unit with the resistance strain gauge attached. The red color in the figure represents the bending deformation of the resistance strain gauge. The deformation amount of the patch unit can be obtained by the single-arm half-bridge measurement method, as shown in Equation (31).

(31) $$\frac{\Delta W_{0}}{W_{0}}=\frac{\Delta L}{L}=\varepsilon$$

where $\Delta W_{0}$ and ΔL are the deformation of the patch and resistance strain gauge, respectively.

The equation for calculating the antenna radiation patch width after the reconfigurable microstrip antenna element is as follows:

(32) $$W_{1}=W_{0}\left(1+\varepsilon \right)$$

Therefore, we can calculate the width of the deformed reconfigurable microstrip patch unit through the strain value measured by the static resistance strain gauge, substitute it into the corresponding electromechanical coupling model to calculate the electrical properties of the antenna, and compare and analyze the measured results to verify its correctness.

**Figure 15:**
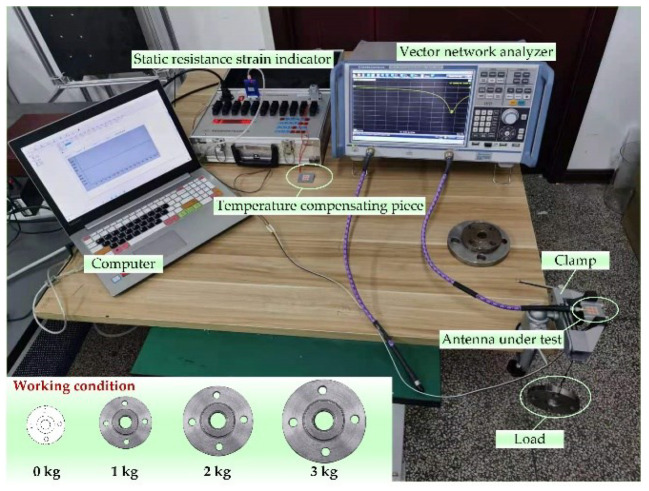
Measurement system.

To achieve different degrees of deformation for the antenna sample, a strain measurement system is designed. The instruments used include resistance strain gauges, static resistance strain indicator, fixtures, flanges, and trays. The designed measurement system is shown in Figure 15, in which flanges of different weights are used to deform the antenna sample to different degrees, and then the strain of the antenna when the structure is deformed is measured by the static resistance strain gauge. Then, the size change of the radiation patch on the surface of the antenna sample is obtained, the electrical properties of the relevant antennas are measured, and the corresponding data are recorded.

### 3.2. Test and Verification of the Resonant Frequency Coupling Model

**Figure 16:**
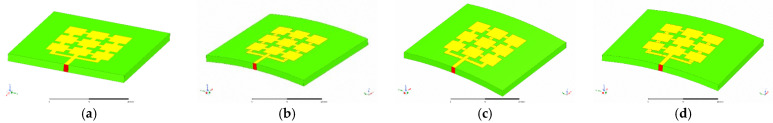
Four working conditions of reconfigurable microstrip antenna deformation measurement. (**a**) Working condition 0; (**b**) working condition 1; (**c**) working condition 2; (**d**) working condition 3.

**Figure 17:**
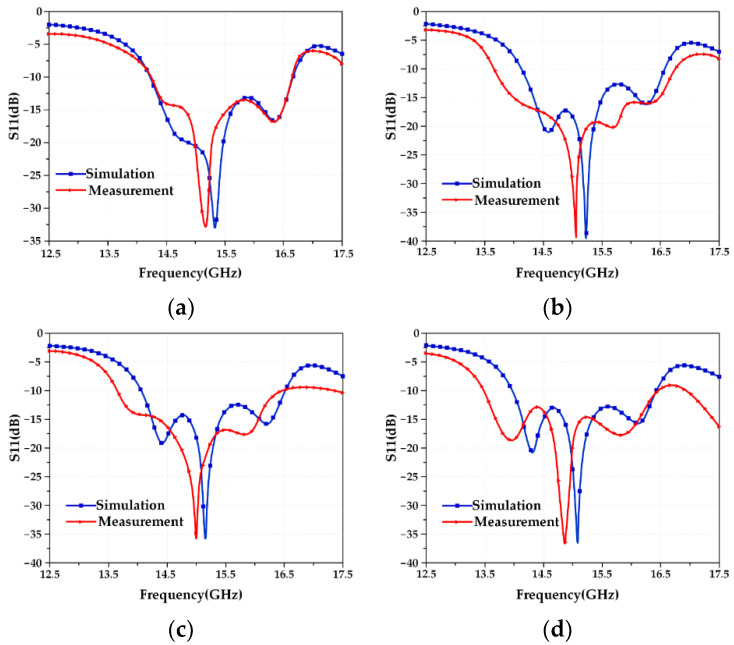
S11 curves of the Mode 1 antenna under four working conditions. (**a**) Working condition 0; (**b**) working condition 1; (**c**) working condition 2; (**d**) working condition 3.

**Figure 18:**
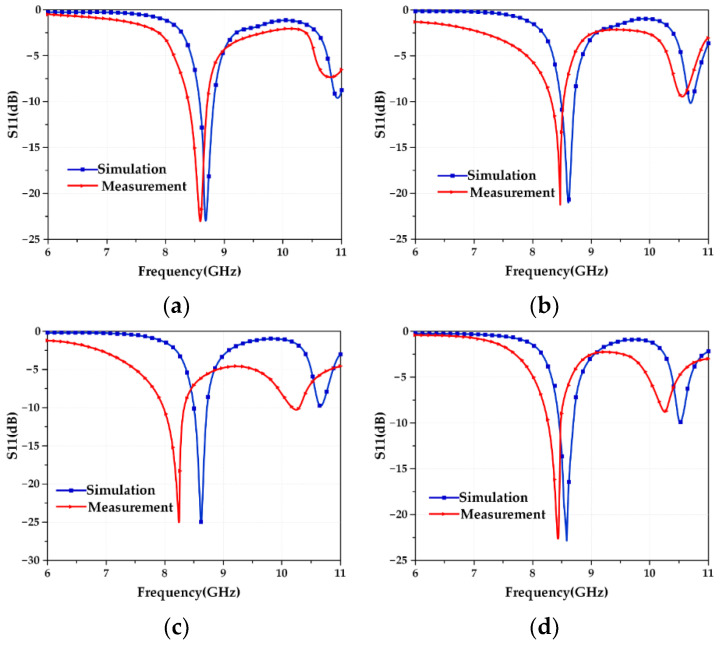
S11 curves of the Mode 2 antenna under four working conditions. (**a**) Working condition 0; (**b**) working condition 1; (**c**) working condition 2; (**d**) working condition 3.

To fully study the perturbation mechanism of the frequency characteristics of the reconfigurable microstrip antenna, this summary gives the test results of the antenna frequency performance under four working conditions. In the HFSS software, the microstrip array antenna models corresponding to the four working conditions are established, as shown in Figure 16. Since the four designed modes of antennas have similar structures, only the electromagnetic model of the Mode 2 antenna is given here as an example, and the simulation calculation is carried out. At the same time, the calculation results of the structural parameter–resonant frequency coupling model are given, and then the S11 test is performed on the Mode 1 and Mode 2 antenna samples under each working condition, and the test results are recorded. Finally, the three are compared and analyzed to verify the coupling of the aircraft. The correctness of the model is shown in Figure 17 and Figure 18, and the results are recorded as shown in Table 14 and Table 15.

Table 14 and Table 15 show that the deviations between the data obtained from the actual test under the four working conditions and the results calculated by the established structural parameter–resonant frequency coupling model are all within 0.1 GHz, and the error does not exceed 5%, which meets general engineering needs. The reasons for the error mainly include two aspects: one is the influence of welding, that is, the solder at the SMA joint will affect the discontinuity of the surface current, and the other is the matching problem of the SMA joint, that is, an ideal port feeding is used in the simulation, and reflections from actual SMA connectors are not considered. Although there is an error, the comparison of the data obtained by the simulation in Figure 17 and Figure 18 shows that the trend of the S11 curves of the two is completely consistent, and the errors are all within 5%, which is a good proof of the validity of the established structural parameter–resonant frequency coupling model in the applicable range.

### 3.3. Test and Verification of Pattern Function Coupling Model

**Figure 19:**
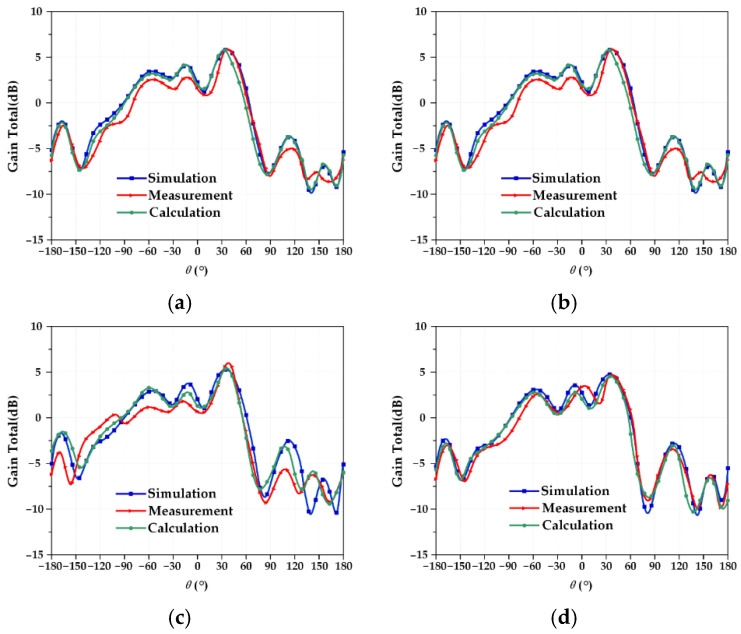
Mode 3 antenna radiation pattern under four working conditions. (**a**) Working condition 0; (**b**) working condition 1; (**c**) working condition 2; (**d**) working condition 3.

**Figure 20:**
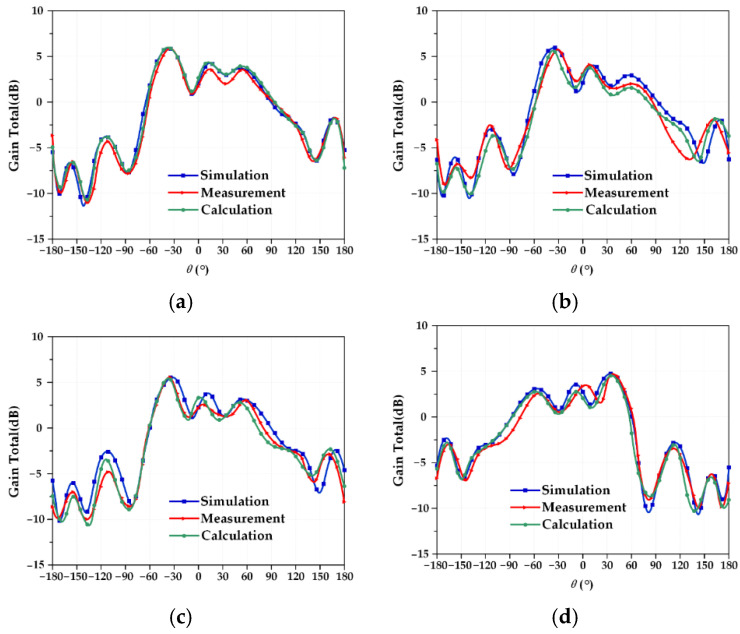
Mode 4 antenna radiation pattern under four working conditions. (**a**) Working condition 0; (**b**) working condition 1; (**c**) working condition 2; (**d**) working condition 3.

To fully study the perturbation mechanism of the frequency characteristics of the reconfigurable microstrip antenna, the actual measurement results under four different working conditions should be compared with the established electromechanical coupling calculation results to verify the structural parameter–resonant frequency coupling model. Due to the particularity of the antenna pattern, it is impossible to measure the pattern of the microstrip antenna while applying an external force to deform the microstrip antenna. Therefore, in this part of the experiment, only the power pattern of the reconfigurable microstrip antenna designed under ideal conditions is tested. The Mode 3 and Mode 4 antennas are measured for the pattern, and the test results are shown in Figure 19 and Figure 20. At the same time, the resonant frequency of the corresponding deformation is calculated by the structural parameter–pattern function coupling model, and the results are recorded, as shown in Table 16 and Table 17.

In Table 16 and Table 17, it can be seen that the maximum error between the data obtained from the actual test under ideal conditions and the result calculated by the established structural parameter–direction diagram coupling model does not exceed 5%. The reasons for the error mainly include two aspects: one is the influence of welding, that is, the solder at the SMA joint will affect the discontinuity of the surface current, which will affect its radiation characteristics; the second is the influence of the angle of the microstrip antenna in the test, which may be fully tuned for a standard horn receiver antenna, resulting in a pattern shift. Although errors occur in the actual test, the maximum error does not exceed 5%, and Figure 19 and Figure 20 show that the patterns of the two antennas are almost completely symmetrical and are very close to the calculation results of the established electromechanical coupling model, which fully verifies the validity of the established structural parameter–direction diagram function coupling model in the applicable range.

### 3.4. Discussion of Results

After rigorous theoretical analysis, simulation analysis, and actual test analysis, the accuracy and effectiveness of the structural parameter-resonant frequency coupling model and the structural parameter-pattern function coupling model of the reconfigurable microstrip antenna proposed in this paper are fully verified. Within the range, the calculation errors are all within 5%, which meets the needs of engineering applications. For a reconfigurable microstrip antenna in different switching states, only one full-wave simulation is needed, and then the equivalent current length, excitation mode, and coupling coefficient of each patch unit are obtained based on the current distribution indicated by the antenna. Then, only the equivalent length and the resonant frequency calculation model of the antenna in this state can be obtained by a slight correction of the width. Then, the pattern function coupling model of the antenna in this state can be obtained based on the obtained excitation mode and coupling coefficient. Based on the established antenna electrical performance coupling model, the electrical performance of the deformed antenna can be quickly predicted, and the solution speed is much faster than that of full-wave simulation, which can provide requirements and theoretical guidance for the design accuracy of an antenna structure and manufacturing errors in engineering.

When designing the frequency or pattern reconfigurable antenna, the hardware configuration used is Intel Core I5-4460 3.20 GHz, 8 G memory, and the development environment is Matlab2021 and HFSS 2021 R1. As can be seen from Table 18, it takes at least 312.8 s to use ANSYS HFSS software to calculate the electrical performance of frequency and pattern reconfigurable antenna, while the calculation time using coupling model is negligible and the calculation efficiency is greatly improved.

The electromechanical coupling model of the reconfigurable microstrip antenna in a complex environment involves the integration of disciplines such as structure, electromagnetism, and materials. When the deformation of the reconfigurable antenna increases, the error of the established electromechanical coupling model also increases. There are two main reasons for this error. One is that we use the idea of equivalent structural deformation to establish the corresponding electromechanical coupling model. If the structural deformation is too large, this equivalent deformation will inevitably fail, so it is only applicable within a certain structural deformation. The second aspect is that after the antenna structure is deformed, the coupling effect between the surface patches is changed. Therefore, using the coupling coefficient before deformation to solve the excitation current will lead to errors. Therefore, in follow-up research, we can obtain more effective structural deformation equivalent methods and obtain the coupling coefficients under different deformations in real time, which will effectively improve the calculation accuracy of the electromechanical coupling model and better meet the real-time requirements of the communication system for the antenna.

## 4. Conclusions

To quickly calculate the electrical performance of reconfigurable microstrip antennas in real time, a fast prediction method of the resonant frequency and the radiation pattern of reconfigurable antenna are proposed in this paper. Then the structural deformation of reconfigurable microstrip antenna in service environment is analyzed and an equivalent model of antenna structural deformation is established. The frequency characteristics and radiation performance of reconstructed antenna can be quickly predicted when structural deformation occurs in the antenna operation. The frequency and radiation performance of reconfigurable antenna can be quickly predicted when structural deformation occurs in the antenna operation. Finally, the experimental verification is carried out and the influence of antenna structure parameters on the antenna electrical performance is analyzed in a certain range. The structural parameters of the resonant frequency coupling model change within 1 mm, and the relative error between the calculation and simulation is less than 5%. The *W*_0_ of the pattern coupling model changes within 0.4 mm, *L*_0_ changes within 0.1 mm, and the relative error between the calculation and simulation is less than 5%. The two coupling models can quickly analyze the changes in antenna electrical performance under the influence of different environmental loads, evaluate the performance of reconfigurable antennas, further guide the structural design of reconfigurable antennas, and provide a theoretical basis for the evaluation of antenna structural design schemes in practical work.

## Figures and Tables

**Table 1:** Antenna structural parameters.

Parameters	Value (mm)	Parameters	Value (mm)	Parameters	Value (mm)
*L*_0_	5	*W*_0_	5	*L_sub_*	32.5
*W_sub_*	32.5	*L_m_*	1.25	*W_m_*	1.25
*L*_02_	6.25	*W*_02_	1.25	*L*_01_	5
*W*_01_	1.25	*h*	2		

**Table 2:** Design results of frequency and pattern reconfigurable antennas.

Reconfigurable Antennas	Operating Frequency Bands (GHz)	Resonant Frequencies (GHz)	Beam Pointing (°)	Maximum Return Loss (dB)	HPBW (°)	Gain Total (dB)
Mode1	14.27–16.80	15.26	−38	−33.15	44.68	8.63
Mode2	8.55–8.78	8.66	−39	−23.04	48.54	8.41
Mode3	14.86–15.79	15.3	+36.2	−24.66	41.9	6.22
Mode4	14.86–15.79	15.3	−36.5	−24.47	42.2	6.42

**Table 3:** Comparison between the resonant frequency of the Mode 1 antenna obtained by HFSS simulation and the calculation results of the coupling model.

Parameters	HFSS Simulation	Calculation
Resonant frequency *f*	15.26 GHz	15.16 GHz

**Table 4:** Comparison between the resonant frequency of the Mode 2 antenna obtained by HFSS simulation and the calculation results of the coupling model.

Parameters	HFSS Simulation	Calculation
Resonant frequency *f*	8.66 GHz	8.86 GHz

**Table 5:** Excitation current of the patch element.

**Patch number**	1	2	3	4	5	6	7	8
**Current (A)**	0.02	0.2	0.4	0.5	0.3	0.01	1	1

**Table 6:** HFSS simulation results and coupling model calculation results of different *W*_0_ and *L*_0_ parameters under Mode 1 antenna.

*L*_0_ (mm) (*W*_0_ = 5 mm)	HFSS Simulation *f* (GHz)	Model Calculation *f* (GHz)	*W*_0_ (mm) (*L*_0_ = 5 mm)	HFSS Simulation *f* (GHz)	Model Calculation *f* (GHz)
4.5	15.98	16.23	4.5	15.94	15.23
4.6	15.82	16.08	4.6	15.88	15.22
4.7	15.66	15.84	4.7	15.82	15.21
4.8	15.52	15.61	4.8	15.76	15.19
4.9	15.40	15.38	4.9	15.72	15.18
5.0	15.26	15.16	5.0	15.26	15.16
5.1	15.52	14.95	5.1	15.16	15.15
5.2	15.30	14.74	5.2	15.04	15.14
5.3	15.12	14.54	5.3	14.96	15.13
5.4	14.96	14.35	5.4	14.84	15.12
5.5	14.8	14.16	5.5	14.68	15.11

**Table 7:** HFSS simulation results and coupling model calculation results of different *W*_0_ and *L*_0_ parameters under Mode 2 antenna.

*L*_0_ (mm) (*W*_0_ = 5 mm)	HFSS Simulation *f* (GHz)	Model Calculation *f* (GHz)	*W*_0_ (mm) (*L*_0_ = 5 mm)	HFSS Simulation *f* (GHz)	Model Calculation *f* (GHz)
4.5	9.24	9.67	4.5	8.82	8.90
4.6	9.12	9.50	4.6	8.80	8.89
4.7	9.0	9.33	4.7	8.76	8.89
4.8	8.88	9.17	4.8	8.72	8.88
4.9	8.80	9.02	4.9	8.72	8.87
5.0	8.66	8.86	5.0	8.66	8.86
5.1	8.58	8.72	5.1	8.64	8.86
5.2	8.45	8.58	5.2	8.62	8.85
5.3	8.38	8.44	5.3	8.58	8.85
5.4	8.26	8.32	5.4	8.56	8.84
5.5	8.16	8.19	5.5	8.52	8.83

**Table 8:** HFSS simulation results and coupling model calculation results of different *W*_0_ and *L*_0_ parameters under Mode 3 antenna.

*L*_0_ (*W*_0_ = 5 mm)	Beam Pointing (°)		*W*_0_ (*L*_0_ = 5 mm)	Beam Pointing (°)	
	HFSS Simulation (°)	Model Calculation (°)		HFSS Simulation (°)	Model Calculation (°)
4.5	31.8	35.5	4.5	31.9	34.1
4.6	32.1	35.6	4.6	32.7	34.5
4.7	33.3	35.7	4.7	34	34.9
4.8	33.3	35.8	4.8	34.6	35.2
4.9	34.9	35.9	4.9	35.4	35.6
5.0	36.2	36	5.0	36.2	36
5.1	37.6	36.1	5.1	36.8	36.4
5.2	40.3	36.2	5.2	38.1	36.8
5.3	42.7	36.3	5.3	36.7	37.2
5.4	−11.6	36.4	5.4	37	37.6
5.5	−9.4	36.5	5.5	36.7	38

**Table 9:** Comparison between model calculation and HFSS simulation results when parameters of *W*_0_ change.

***W*** **_0_** **(mm)**	4.5	4.6	4.7	4.8	4.9	5.0	5.1	5.2	5.3	5.4	5.5
**Error (°)**	2.2	1.8	0.9	0.6	0.2	0.2	0.4	1.3	0.5	0.6	1.3
**Relative error (%)**	6.9	5.5	2.6	1.7	0.5	0.5	1	3.4	1.3	1.6	3.5

**Table 10:** Comparison between model calculation and HFSS simulation results when parameters of *L*_0_ change.

***L*** **_0_** **(mm)**	4.5	4.6	4.7	4.8	4.9	5.0	5.1	5.2	5.3	5.4	5.5
**Error (°)**	3.7	3.5	2.4	2.5	1	0.2	1.5	4.1	6.4	48	45.9
**Relative error (%)**	11.6	10.9	7.2	7.5	2.8	0.5	3.9	10.1	14.9	-	-

**Table 11:** HFSS simulation results and coupling model calculation results of different *W*_0_ and *L*_0_ parameters under Mode 4 antenna.

*L*_0_ (*W*_0_ = 5 mm)	Beam Pointing (°)		*W*_0_ (*L*_0_ = 5 mm)	Beam Pointing (°)	
	HFSS Simulation (°)	Model Calculation (°)		HFSS Simulation (°)	Model Calculation (°)
4.5	27.2	−36	4.5	−31.4	−34.5
4.6	−32.3	−36.1	4.6	−33.7	−34.9
4.7	−33.1	−36.2	4.7	−35.1	−35.3
4.8	−33.7	−36.2	4.8	−34.3	−35.6
4.9	−36.5	−36.3	4.9	−35.7	−36
5.0	−36.5	−36.4	5.0	−36.5	−36.4
5.1	−37.9	−36.4	5.1	−36.8	−36.7
5.2	−39.7	−36.5	5.2	−37.9	−37.1
5.3	−43	−36.6	5.3	−37.3	−37.5
5.4	12.8	−36.6	5.4	−35.8	−37.9
5.5	10.1	−36.7	5.5	−36	−38.2

**Table 12:** Comparison between model calculation and HFSS simulation results when parameters of *W*_0_ change.

***W*** **_0_** **(mm)**	4.5	4.6	4.7	4.8	4.9	5.0	5.1	5.2	5.3	5.4	5.5
**Error (°)**	3.1	1.2	0.2	1.3	0.3	0.1	0.1	0.8	0.2	2.1	2.2
**Relative error (%)**	9.8	3.5	0.5	3.7	0.8	0.2	0.2	2.1	0.5	5.8	6.1

**Table 13:** Comparison between model calculation and HFSS simulation results when parameters of *L*_0_ change.

***L*** **_0_** **(mm)**	4.5	4.6	4.7	4.8	4.9	5.0	5.1	5.2	5.3	5.4	5.5
**Error (°)**	63.2	3.8	3.1	2.5	0.2	0.1	1.5	3.2	6.4	49.4	46.8
**Relative error (%)**	-	11.7	9.3	7.4	0.5	0.2	3.9	8	14.8	-	-

**Table 14:** Resonant frequencies of the Mode 1 antenna under four working conditions.

Working Condition	Size after Deformation (*W*_0_/mm)	Measurement Results (GHz)	Simulation Results (GHz)	Calculation Results (GHz)
0	5.0	15.15	15.26	15.160
1	5.135	15.12	15.21	15.143
2	5.236	15.01	15.18	15.131
3	5.338	14.92	15.15	15.120

**Table 15:** Resonant frequencies of the Mode 2 antenna under four working conditions.

Working Condition	Size after Deformation (*W*_0_/mm)	Measurement Results (GHz)	Simulation Results (GHz)	Calculation Results (GHz)
0	5.0	8.55	8.66	8.861
1	5.131	8.51	8.63	8.852
2	5.234	8.37	8.59	8.856
3	5.336	8.49	8.57	8.840

**Table 16:** Maximum radiation direction of the Mode 3 antenna under four working conditions.

Working Condition	Size after Deformation (*W*_0_/mm)	Measurement Results (°)	Simulation Results (°)	Calculation Results (°)
0	5.0	36.18	36.20	36.20
1	5.132	36.52	36.63	36.45
2	5.233	36.69	36.78	36.82
3	5.337	37.21	37.12	37.26

**Table 17:** Maximum radiation direction of the Mode 4 antenna under four working conditions.

Working Condition	Size after Deformation (*W*_0_/mm)	Measurement Results (°)	Simulation Results (°)	Calculation Results (°)
0	5.0	−36.48	−36.50	−36.4
1	5.133	−36.71	−36.66	−36.76
2	5.232	−37.12	−37.03	−37.23
3	5.336	−37.36	−37.15	−37.65

**Table 18:** Comparison between the computation time by HFSS simulation and the coupling model calculation.

Reconfigurable Antennas	HFSS Simulation	Coupling Model Calculation
Mode 1 Antenna	352.2 s	0.026 s
Mode 2 Antenna	324.6 s	0.029 s
Mode 3 Antenna	321.5 s	0.880 s
Mode 4 Antenna	312.8 s	0.920 s
